# Why do sports goods manufacturers choose different corporate social responsibility engagements?

**DOI:** 10.1371/journal.pone.0295682

**Published:** 2023-12-21

**Authors:** Yang Guo, Dongliang Kang, Chengyin Huang, Ying Chen

**Affiliations:** 1 Department of Network Security, Henan Police College, Zhengzhou, Henan, China; 2 School of E-commerce and Logistics Management, Henan University of Economics and Law, Zhengzhou, Henan, China; 3 College of Physical Education, Chongqing University, Chongqing, China; University of Naples Federico II: Universita degli Studi di Napoli Federico II, ITALY

## Abstract

This study examines the impact of financialization and product market competition on the corporate social responsibility (CSR) engagements in sports goods manufacturing industry. Utilizing a dataset of China’s listed firms, we employ textual analysis to identify organizations within this sector and create a panel data model to analyze the determinants of CSR engagements. Our empirical findings reveal that financialization and product market competition positively influence shareholder-related CSR engagements. Additionally, product market competition enhances the effect of financialization on these engagements. Conversely, a negative correlation exists between product market competition and stakeholder-related CSR engagements. Moreover, firms not categorized as State-Owned Enterprises (SOEs) or within high-pollution industries show a positive response in CSR engagements to both financialization and product market competition. Our results also highlight that managerial compensation and financial constraints modify the impacts of financialization and product market competition on shareholder-related CSR engagements. Collectively, our findings shed light on the challenges that sports goods manufacturing firms face in aligning their primary goals with CSR commitments.

## Introduction

Manufacturing operations significantly contribute to environmental degradation, leading to various social challenges [1]. To mitigate these impacts, governments worldwide have implemented focused on reducing emissions and improving energy efficiency in manufacturing. However, constrain profitability, a tension especially evident in developing economies reliant on manufacturing for economic growth [2, 3]. China, as the world’s largest energy consumer and second-largest economy, China exemplifies this challenge. Balancing economic and environmental benefits has become crucial for sustainable development in emerging economies. Within this context, Corporate Social Responsibility (CSR) emerges as a mechanism to balance these competing interests [4–6]. CSR requires firms to integrate societal well-being and environmental conservation into their profit-maximization strategies. For instance, Adidas, renowned for its commitment to sustainability and social responsibility, has significantly reduced carbon emissions, water usage, and waste. Practically, CSR engagements represent the outcomes of firms’ investment in CSR, reflecting their efforts towards societal betterment and environmental protection [7]. Therefore, CSR engagements in manufacturing offer a promising avenue to reconcile the conflict between environmental preservation and economic growth.

**Fig 1:**
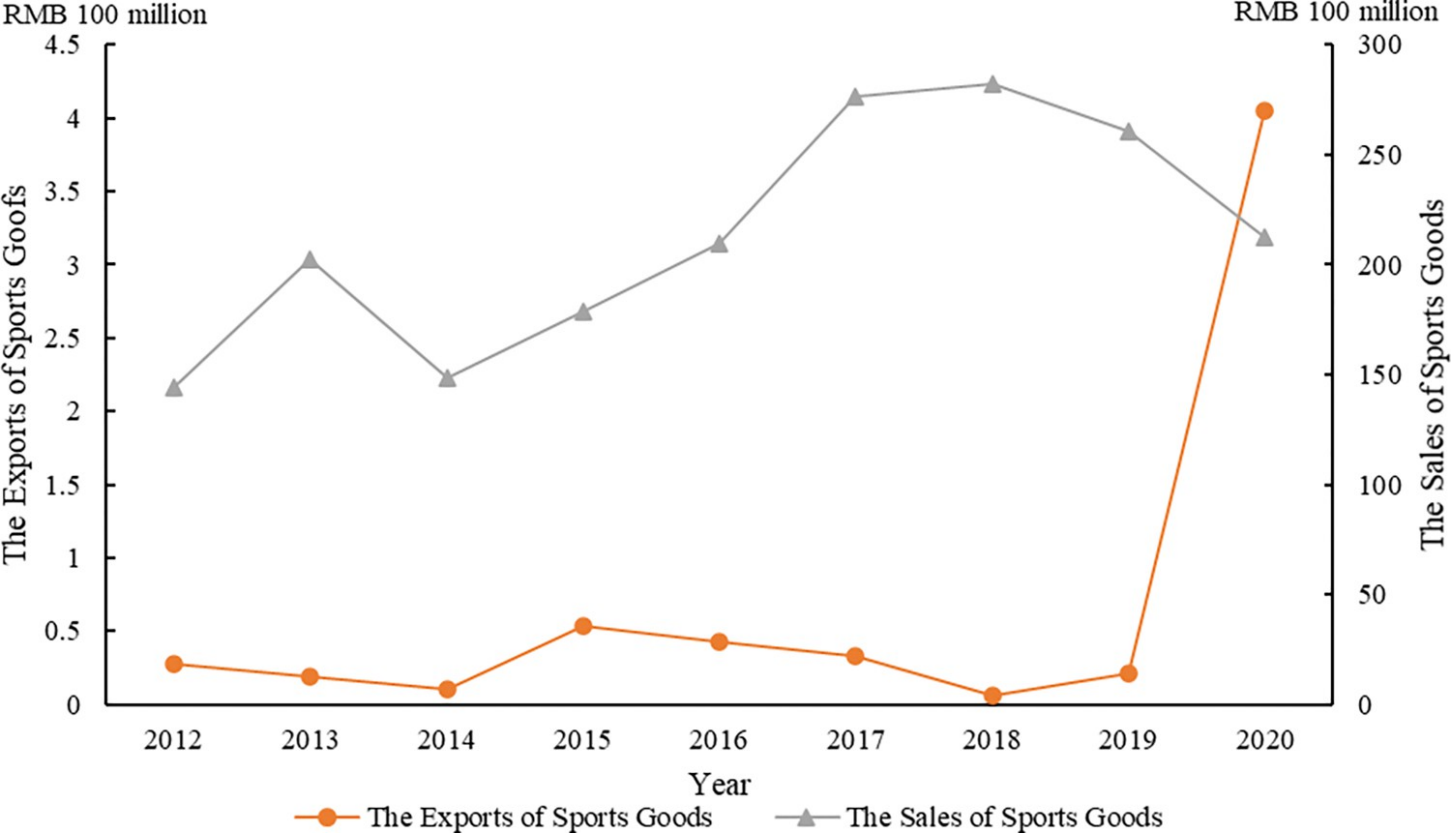
The exports and sales of sports goods in China.

Aligned with CSR principles, manufacturing firms must balance societal well-being and environmental sustainability with shareholder value maximization [8]. Manufacturing inherently poses environmental risks, such as air and water pollution. Accordingly, CSR engagements serve as a viable avenue to alleviate the friction CSR engagements offer a means to mitigate the tension between ecological conservation and economic goals [3]. Sports goods manufacturers, a specific segment within the manufacturing sector, are generally smaller and less profitable but produce goods with substantial impact, especially in emerging economies [9, 10]. Fig 1 shows an increase in domestic demand for sports goods in China until 2018, followed by a decline during the 2019–2020 COVID-19 pandemic. However, China’s sports goods exports rose post-2019, reflecting continued global demand. This demand surge may exacerbate environmental issues like air and water pollution, and energy wastage in the sports goods sector [11, 12]. Adopting eco-friendly practices is thus crucial for these firms to meet societal expectations and maintain innovation. From a resource-based view, sports goods manufacturers engage in CSR primarily to satisfy societal demands or boost shareholder value. The challenge for scholars and practitioners lies in understanding the distinct CSR strategies of sports goods manufacturers.

Adhering to CSR principles, sports goods manufacturers must navigate a complex environment shaped by societal expectations and conflicts between shareholders and stakeholders, within distinct institutional contexts [13, 14]. These firms largely depend on external financial support, heightening the influence of shareholder interests on their CSR strategies [10, 15]. Managerial focus often shifts toward short-term financial investments, potentially neglecting long-term sustainability commitments, a trend termed financialization [16, 17]. Moreover, the substitutability of sports goods creates intense market competition, forcing managers to address stakeholder needs while striving for competitive advantages and superior performance [18]. Consequently, financialization and market competition become critical factors in shaping sports goods manufacturers’ CSR strategies.

This study uses a textual analysis method to identify relevant firms listed on the Shanghai and Shenzhen Stock Exchanges, focusing on CSR engagements within the sports goods manufacturing sector. We categorize CSR activities into shareholder-related and stakeholder-related engagements. Using panel data from China’s listed firms, we investigate the determinants of these CSR engagements. Our empirical findings reveal that: (1) both financialization and market competition positively influence shareholder-related CSR engagements, with market competition enhancing financialization’s impact. Conversely, there’s a negative relationship between market competition and stakeholder-related CSR engagements; (2) ownership structure influences CSR engagements, with non-SOEs encouraged and SOEs deterred from stakeholder-related activities by financialization and market competition; (3) in high-polluting industries, market competition influences the CSR decisions of non-high-polluting firms, whereas financialization reduces high-polluting firms’ focus on stakeholder interests; (4) severe financial constraints alter the effect of financialization and market competition on shareholder-related CSR engagements; and (5) managerial compensation serves as an additional factor incentivizing shareholder well-being, thereby mitigating agency problems among sports goods manufacturers.

To enhance the methodological rigor and theoretical contributions of this study, we offer these insights: First, we illuminate the tensions between shareholder and stakeholder interests in the sports goods manufacturing sector, revealing a tendency to prioritize shareholder welfare over environmental protection. This sheds light on the intricate balance between economic and environmental benefits. Second, our research shows how industry-specific market boundaries significantly impact CSR decisions, with business models and product attributes being key factors. Lastly, our results suggest that sports goods manufacturers in China may not fully meet their intended role, potentially hindering the sustainable progress of the larger economy.

## Literature review and hypotheses

### CSR engagements

CSR engagements, based on CSR principles, specifically focus on corporate practices related to environmental protection and the fulfilment of societal expectations [8]. While the primary goal of CSR is to encourage ethical integration into business operations, some firms view CSR engagements merely as strategies to enhance financial performance, overlooking shareholder interests [19]. This can lead to conflicts between shareholders and stakeholders due to varying CSR engagements [20].

An increasing number of firms are disclosing their societal and environmental actions, motivated by the desire to gain trust from investors who favor responsible management, as explained by the legitimacy theory. Studies have demonstrated a close relationship between corporate social responsibility and financial performance [8, 21–23]. Firms can strategically utilize CSR to achieve their development goals, enhance their environmental performance, and strengthen their reputation and innovation capacity [24–26]. On the other hand, some firms invest heavily in green technology to achieve sustainable development objectives alongside their partners. Considering the well-being of stakeholders, Hillman and Keim explained that firms with better stakeholder relations benefit shareholders [27]. Furthermore, stakeholder-related CSR activities can contribute to firms’ positive reputations [28–30]. Conflicts between shareholders and stakeholders can have negative impacts on the sustainable development of firms, highlighting the importance of implementing appropriate CSR initiatives that balance economic and environmental benefits [21].

### Financialization and CSR engagements

Financialization, while beneficial for short-term revenue, can be detrimental to long-term economic development [31]. According to agent theory, short-term financial gains can appease shareholders and reduce conflicts between managers and owners [32]. However, an overemphasis on financial assets can alter firms’ capital structure and increase their exposure to potential risks. The updates of products or technology are essential for promoting the long-term development of manufacturing firms [33]. Prioritizing financial assets over R&D activities to maximize profits may hinder firms’ long-term development potential [34].

Financialization can enable managers to meet the expectations of shareholders, potentially leading to the adoption of CSR engagements that benefit shareholders [32]. Financialization can maximize shareholder value in certain large digital platforms, provided that there are adequate organizational resources [35]. Some financial assets can also relieve the pressure of mergers and acquisitions faced by managers, especially in manufacturing industries [36]. However, financialization may also pose risks by diverting managers’ attention to short-term interests, which can compromise the long-term sustainability of firms. Destek and Manga explored the impact of financialization on environmental quality, and found that financialization may cause some environmental problems [37]. Additionally, having more financial assets may make firms less inclined to take risks and urge managers to prioritize short-term investments [16]. Therefore, this study proposes the following hypothesis:

**Hypothesis 1a (H1a):** There is an association between financialization and shareholder-related CSR engagements.
**Hypothesis 1b (H1b):** There may be no association between financialization and stakeholder-related CSR engagements.

### Product market competition and CSR engagements

The competitive environment in which firms operate can have a significant impact on their profitability, as managers are compelled to devise effective strategies to maintain their competitiveness [38]. In order to gain a competitive edge in the market, manufacturing firms must rely on their products [9], which ultimately connect them with consumers and suppliers, who are considered stakeholders [39].

According to the resource-based theory, firms face limitations in their ability to allocate resources towards their development strategies [40]. Competition can hinder certain corporate practices, particularly those related to environmental, social, and governance factors [41]. Zou et al. pointed out that industrial competition could promote corporate environmental performance, and also strengthen the impact of managers’ compensation on environmental management [18]. Firms operating in competitive markets are more likely to adopt CSR initiatives to meet society’s expectations [42]. When faced with conflicts between shareholders and stakeholders, competition can incentivize managers to prioritize shareholder welfare, potentially neglecting stakeholder interests [43, 44]. Therefore, this study proposes the following hypothesis:

**Hypothesis 2a (H2a):** There is an association between product market competition and shareholder-related CSR engagements.
**Hypothesis 2b (H2b):** There is an association between product market competition and stakeholder-related CSR engagements.

### Financialization, product market competition and CSR engagements

Managers may select CSR engagements based on short-term or long-term profit preferences [20]. Tsendsuren et al. examined the relationship between product market competition, managerial competency, and environmental management, and discovered that these factors are closely related [45]. Managers with lower levels of managerial competency may be inclined to invest in financial assets to demonstrate their capabilities under the pressure of product market competition [46]. Financialization can be viewed as an essential tool for managers to achieve higher performance evaluations during competitive periods, which, in turn, will enhance the welfare of shareholders [32]. Conversely, managers in less competitive firms may allocate more resources to financial assets, meeting shareholder expectations but potentially exacerbating shareholder-stakeholder conflicts [47]. Therefore, this study proposes the following hypothesis:

**Hypothesis 3 (H3):** The association between financialization and shareholder-CSR engagements will be strengthened by product market competition.

## Research design

### Data sources

This study selects China’s listed manufacturing firms in the Shanghai Stock Exchange and the Shenzhen Stock Exchange as research samples. Sports goods manufacturing firms cannot be labelled by the traditional industry classification standards, especially in the Chinese market. This study adopts the method based on textual analysis to identify sports goods manufacturing firms [9]. Based on sports goods words, this study selects the listed manufacturing firms providing sports goods from 2010 to 2018 as research samples. Some samples falling into one of these categories are excluded: (1) samples with the missing data of variables; (2) samples that are listed for less than 5 years; (3) samples whose MD&A section in annual reports cannot be transformed into the txt file format.

The data of CSR engagements are obtained from the Hexun CSR Database, whose score is widely used to evaluate the CSR activities of China’s listed firms [48]. The data of corporate finance and government are mainly derived from the China Stock Market and Accounting Research Database (CSMAR). Moreover, the annual reports of research samples are collected from the website of the Shanghai Stock Exchange and the Shenzhen Stock Exchange. For minimizing the impact of extreme values in regression analysis, all continuous variables are winsorized at 1% at both tails.

### Variables

#### Dependent variable

CSR engagements represent firms’ CSR activities during their business operations. Considering the conflict between shareholders and stakeholders, managers need to adopt different CSR activities to maximize the benefit of owners or meet the expectations of the society [20]. In this situation, CSR engagements can be regarded as a strategic tool to help firms achieve their goals of development [8]. According to Hillman and Keim (2001), most firms have to balance the relationship between stakeholder management and shareholder value, which becomes a critical factor in promoting the sustainability of those firms. On the one hand, some shareholder-related CSR activities, which can be called strategic CSR, can help firms to strengthen their market position and increase profits [19]. On the other hand, some stakeholder-related CSR activities will enhance customer loyalty and employee productivity, which may improve the financial performance of firms [49]. Referring to Havlinova and Kukacka (2021), this study uses the stakeholders-related and shareholders-related activities to represent different CSR engagements.

#### Independent variables

Financialization represents the financialization of listed firms, which can be measured by firms’ investments in financial assets. Faced with the pressure of shareholders, managers have to improve the profitability of firms in the short run, which can distract manufacturing firms from their intended purpose. Based on Tori and Onaran (2018) [50], some specific assets related to financial and real estate industries can measure the financialization of firms. Therefore, the proportion of financial assets in total assets will be used to measure the financialization of sports goods manufacturing firms.

Competition represents product market competition faced by sports goods manufacturing firms. This study diverges from traditional competition measurement methods and instead employs a textual analysis approach to construct an individual firm’s competitive environment in the sports goods manufacturing sector [51]. The core idea of this method is similar to Text-based Network Industry Classifications (TNIC) proposed by Hoberg and Phillips in 2016, who used the product-related words to identify the boundaries of product market [52]. Referring to Huang and Chen (2022) and Lee et al. (2015) [53], the following steps are used to identify the rivals of each firm, and measure their competition degree:

Step 1. **Word Collection:** The words of sports goods are collected from the files of industry classification systems and the annual reports of A-share listed firms. In this step, these words are extracted from China’s National Sports Industry Classification, the International Standard Industrial Classification (ISIC) published by United Nations. Moreover, some words are obtained from the annual reports through manual screening.

Step2. **Firm Identification:** This study uses the words of sports goods to identify sports goods manufacturing firms from A-share listed firms, who mentioned sports goods in the MD&A section of their annual reports.

Step3. **Word Vector Construction:** After identifying sports goods manufacturing firms, the method of “Total Frequency-Inverse Document Frequency” (TF-IDF) is used to construct the word vector of firm i based on the words of sports goods.

Step 4. **Product Similarity Computation:** For firm *i*, cosine similarity function is chosen to compute the product similarity between firm i and other firms in year t.

Step 5. **Rival Identification and Competition Measurement:** Based on the similarity results of firm *i*, this study identifies the 10 most similar firms as its rivals, and uses the sum of rivals’ similarities to measure the competition degree of firm *i*.

#### Control variables

We control the impact of other dimensions on CSR engagements, including financial dimension, governance dimension and government dimension [20, 29, 43]. Our control variables include firm size, operation revenues, largest shareholder, independent directors, board size, dual occupancy, and government subsidies. Firm size (Size) represents the total assets of firm. Operation revenues (Sale) represents the profitability of firm. The largest shareholder (First) represents the shareholding structure of firm. Independent directors (Independ) and board size (Director) represent the governance structure of the firm. Dual is a dummy variable, and represents whether the chairman and CEO of the firm are the same person. Government subsidies (Subsidy) represent the local government support for sports goods manufacturing firms. The definitions of all variables are reported in Table 1.

**Table 1:** The definitions of all variables.

Variables	Type	Definition
CSR_Shareholder	Dependent Variables	CSR_Shareholder is measured as the score of shareholder-related activities from Hexun CSR Database.
CSR_Stakeholder		CSR_Stakeholder is measured as the score of stakeholder-related activities from Hexun CSR Database.
Fin	Independent Variables	Fin represents corporate financialization, which is calculated as the financial assets divided by the total assets.
Competition		Competition represents sports goods market competition, which is measured by the similarity between focal firm and its peer firms.
Size	Control Variables	Size represents firm size, which is measured by the natural logarithm of the firm’s total assets.
Sales		Sales represents operation revenues, which is calculated as the growth of operation revenues.
First		First represents the largest shareholder, which is calculated as the shareholding ratio of the largest shareholder.
Independ		Independ represents independent directors, which is calculated as the number of independent directors divided by the total number of directors on the board.
Director		Director represents board size, which is measured by the natural logarithm of the total number of directors.
Dual		Dual represents dual occupancy, which will be 1, if the chairman and CEO of the firm are the same person, or otherwise 0.
Subsidy		Subsidy represents government subsidies, which is calculated as the government subsidies divided by the firm’s total assets.

#### Empirical models

In order to explore the driving factors of CSR engagements, we construct two empirical models to discuss the association among financialization, competition and CSR engagements (shareholder-related activities and stakeholder-related activities). In the first empirical model, we will explore the impact of financialization and competition on CSR engagements. In the second empirical model, the interaction of financialization and competition will be introduced into the first model, and this model will discuss the impact of competition on the association between financialization and CSR engagements. The first empirical model is as follows:

$${CSR\;Engagements}_{i,t}=\alpha_{0}+\beta_{1}{Fin}_{i,t}+\beta_{2}{Competition}_{i,t}+\beta_{3}{Size}_{i,t}+\beta_{4}{Sales}_{i,t}+\beta_{5}{First}_{i,t}+\beta_{6}{Independ}_{i,t}+\beta_{7}{Director}_{i,t}+\beta_{8}{Dual}_{i,t}+\beta_{9}{Subsidy}_{i,t}+{Year}_{fixed}+{Industry}_{fixed}+\varepsilon$$ (1)

In Eq (1), *CSR Engagements*_*i*,*t*_ represents the different CSR activities of firm i in year t, including shareholder-related activities *CSR_Shareholder*_*i*,*t*_ and stakeholder-related activities *CSR_Stakeholder*_*i*,*t*_. *Fin*_*i*,*t*_ represents the financialization of firm i in year t. *Competition*_*i*,*t*_ represents the competition degree of firm i in year t. *Size*_*i*,*t*_ represents the total assets of firm i in year t. *Sales*_*i*,*t*_ represents the growth of operation revenue of firm i in year t. *First*_*i*,*t*_ represents the shareholding ratio of largest shareholder of firm i in year t. *Independ*_*i*,*t*_ represents the proportion of independent directors of firm i in year t. *Director*_*i*,*t*_ represents the board size of firm i in year t. *Dual*_*i*,*t*_ represents whether the chairman and CEO of firm i in year t is the same person. *Subsidy*_*i*,*t*_ represents the government subsidies of firm i in year t. *Year*_*fixed*_ will control the year fixed effects. *Industry*_*fixed*_ will control the industry fixed effects. *ε* is the error term.


$${CSR\;Engagements}_{i,t}=\alpha_{0}+\beta_{1}{Fin}_{i,t}+\beta_{2}{Competition}_{i,t}+\beta_{3}{Fin}_{i,t}\times {Competition}_{i,t}+\beta_{4}{Size}_{i,t}+\beta_{5}{Sales}_{i,t}+\beta_{6}{First}_{i,t}+\beta_{7}{Independ}_{i,t}+\beta_{8}{Director}_{i,t}+\beta_{9}{Dual}_{i,t}+\beta_{10}{Subsidy}_{i,t}+{Year}_{fixed}+{Industry}_{fixed}+\varepsilon$$ (2)

Eq (2) is the second empirical model. Based on Eq (1), we introduce the interaction of financialization and competition (*Fin*_*i*,*t*_×*Competition*_*i*,*t*_) to test the moderating effect of product market competition. This model will demonstrate whether competition can change the impact of financialization on managers’ preferences for different CSR engagements.

### Empirical results

#### Descriptive statistics

Table 2 reports the descriptive statistical results of all variables in empirical models. For CSR engagements, the median and mean of shareholder-related activities CSR_Shareholder are 14.7600 and 13.9893, indicating that most sports goods manufacturing firms have paid similar attention to these strategic CSR activities. It is worth noting that the maximum of stakeholder-related activities CSR_Stakeholder is 53.8600, which is much higher than the mean or median of this variable (8.6464 and 5.7350), suggesting that there are obvious differences in this CSR engagements among sports goods manufacturing firms. For the financialization of sports goods manufacturing firms, the maximum of Fin (0.6785) is much higher than the mean or median of this variable (0.0996 and 0.0494), indicating that some firms invest too many resources in financial assets, and part of them has been distracted from their intended purpose. For the variable of competition, its median (0.7554) is close to its mean (0.6996), as well as its maximum value (1.0000), suggesting that most sports goods manufacturing firms face a competitive environment.

**Table 2:** The descriptive statistics of all variables.

Variables	Observations	Mean	Std. Dev.	Min	Median	Max
CSR_Shareholder	2116	13.9893	6.3632	-2.8900	14.7600	24.7500
CSR_Stakeholder	2116	8.6464	11.2987	-5.7300	5.7350	53.8600
Fin	2116	0.0996	0.1287	0.0000	0.0494	0.6785
Competition	2116	0.6996	0.2833	0.0000	0.7554	1.0000
Size	2116	21.8457	1.1567	17.6413	21.6861	27.3861
Sales	2116	0.2197	0.6363	-0.7008	0.0872	4.3950
First	2116	0.3433	0.1427	0.0965	0.3275	0.7705
Independ	2116	0.3754	0.0521	0.3333	0.3636	0.5714
Director	2116	2.2300	0.1682	1.7918	2.3026	2.6391
Dual	2116	0.3270	0.4692	0.0000	0.0000	1.0000
Subsidy	2116	0.0032	0.0124	0.0000	0.0000	0.3377

Table 3 reports the Pearson and Spearman correlation coefficients of all variables in empirical models. The correlation coefficients of Fin and CSR engagements are 0.0842 and -0.0330, indicating that the financialization of sports goods manufacturing firms may have different impacts on different CSR activities. In addition, the correlation coefficients of Competition and CSR engagements are 0.0258 and -0.0850, indicating that the competitive environment faced by sports goods manufacturing firms may influence the managers’ adoption of different CSR activities. For control variables, the absolute correlation coefficients of these variables and independent (dependent) variables are less than 0.6, which demonstrates that our empirical models could not be influenced by the collinearity problem. In the light of this, the empirical analysis will provide accurate and practical evidences to research hypotheses.

**Table 3:** The correlation coefficient of all variables.

Variables	CSR_Shareholder	CSR_Stakeholder	Fin	Competition	Size	Sales
CSR_Shareholder		0.3085***	0.0546**	0.0346	0.1016***	-0.0072
CSR_Stakeholder	0.2060***		0.0434**	-0.0957***	0.1976***	0.0204
Fin	0.0842***	-0.0330		0.0083	0.1298***	0.0028
Competition	0.0258	-0.0850***	-0.0188		0.0874***	-0.0141
Size	0.1444***	0.2218***	0.0105	0.0878***		0.0035
Sales	-0.0409*	-0.0174	0.0120	-0.0430**	-0.0113	
First	0.2270***	0.1435***	-0.0603***	-0.0345	0.1569***	-0.0179
Independ	0.0518**	0.0167	0.0021	0.0017	-0.0378*	0.0398*
Director	0.0131	0.1175***	-0.0612***	-0.0123	0.2210***	0.0041
Dual	0.0588***	-0.0861***	-0.0245	-0.0112	-0.1400***	-0.0141
Subsidy	-0.0419*	0.0034	-0.0103	-0.0566***	-0.0744***	0.1206***
**Variables**	**First**	**Independ**	**Director**	**Dual**	**Subsidy**	
CSR_Shareholder	0.2060***	0.0543**	0.0118	0.0734***	-0.0700***	
CSR_Stakeholder	0.1884***	0.0293	0.0587***	-0.0696***	-0.0692***	
Fin	-0.0719***	0.0156	-0.0561***	-0.0232	-0.1093***	
Competition	-0.0249	0.0259	-0.0400*	-0.0112	-0.0235	
Size	0.0924***	-0.0546**	0.2150***	-0.1528***	-0.1408***	
Sales	0.0034	0.0421*	-0.0070	0.0114	0.0176	
First		0.0810***	-0.0570***	0.0131	-0.0330	
Independ	0.1005***		-0.5785***	0.0803***	-0.0032	
Director	-0.0482**	-0.5426***		-0.1582***	-0.0223	
Dual	0.0113	0.0932***	-0.1500***		0.0258	
Subsidy	-0.0133	-0.0119	-0.0270	-0.0001		

Note: ***, **, * represent the significance at the level of 1%, 5%, 10% respectively. The lower triangular matrix reports the Pearson correlation coefficients, and the upper triangular matrix reports the Spearman correlation coefficients.

#### Baseline test

In order to explore the driving factors of CSR engagements, this study uses the panel data models to discuss the impact of financialization and competition on different CSR activities. According to Eq (1), we will test whether financialization or competition can influence the CSR engagements of sports goods manufacturing firms. Furthermore, Eq (2) will be used to examine the impact of competition on the association between financialization and CSR engagements. In the regression, the effect of industry will focus on the manufacturing industries based on the Guidance for Industry Classification of Listed Companies released by the China Securities Regulatory Commission in 2001. The baseline results of shareholder-related CSR engagements are reported in Table 4.

**Table 4:** The baseline results of shareholder-related CSR engagements.

Variables	CSR_Shareholder						
	(1)	(2)	(3)	(4)	(5)	(6)	(7)
Fin	2.9834**		3.0928***	3.9273***		4.0313***	4.1378***
	(2.51)		(2.64)	(3.50)		(3.63)	(3.93)
Competition		0.9265*	0.9991**		0.8416*	0.9370*	0.8863*
		(1.88)	(2.02)		(1.77)	(1.96)	(1.87)
Fin * Competition							13.0361***
							(4.25)
Size				0.6615***	0.6454***	0.6560***	0.6529***
				(5.33)	(5.19)	(5.29)	(5.25)
Sales				-0.5584***	-0.5589***	-0.5472***	-0.5516***
				(-2.70)	(-2.70)	(-2.64)	(-2.66)
First				9.4356***	9.3273***	9.4557***	9.4722***
				(9.95)	(9.82)	(9.98)	(10.02)
Independ				3.4662	2.9530	3.4221	2.7605
				(1.15)	(0.98)	(1.14)	(0.92)
Director				1.5161	1.3609	1.5501*	1.2620
				(1.62)	(1.46)	(1.66)	(1.36)
Dual				0.9129***	0.8574***	0.9248***	0.9087***
				(3.17)	(2.98)	(3.22)	(3.17)
Subsidy				-8.7160	-7.1378	-7.8667	-6.2925
				(-1.15)	(-1.02)	(-1.05)	(-0.91)
Constant	12.0672***	11.7331***	11.5085***	-10.3559***	-9.6677***	-10.8365***	-9.9913***
	(16.83)	(15.18)	(14.72)	(-3.01)	(-2.80)	(-3.14)	(-2.91)
Year	Yes	Yes	Yes	Yes	Yes	Yes	Yes
Industry	Yes	Yes	Yes	Yes	Yes	Yes	Yes
Observations	2116	2116	2116	2116	2116	2116	2116
R^2^	0.0485	0.0467	0.0503	0.1233	0.1188	0.1248	0.1309

Note: ***, **, * represent the significance at the level of 1%, 5%, 10% respectively; t statistics are in parentheses.

In Table 4, Columns (1)—(3) respectively test the impact of financialization and product market competition on the shareholder-related CSR engagements of sports goods manufacturing firms without considering control variables. In Column (1), the coefficient of Fin is 2.9834, significant at the 5% level, indicating that financialization can promote shareholder-related CSR activities. In Column (2), the coefficient of Competition is 0.9265, significant at the 10% level, demonstrating that there is a positive association between product market competition and shareholder-related CSR activities. In Column (3), financialization and product market competition both have positive impacts on shareholder-related CSR activities. After introducing control variables, the results of Columns (4)–(6) support the hypothesis that financialization and product market competition can motivate the managers of sports goods manufacturing firms to pay more attention to shareholder-related CSR activities. Column (7) examines the impact of product market competition on the association between financialization and shareholder-related CSR engagements. Based on the result of Column (7), Competition can significantly strengthen the impact of Fin on CSR_Shareholder, indicating that a competitive environment may change the preference of managers for financial assets, which also forces managers to pay more attention to the well-being of shareholders.

In Table 5, Columns (1)—(3) respectively test the impact of financialization and product market competition on the stakeholder-related CSR engagements of sports goods manufacturing firms without considering control variables. In Column (1), the coefficient of Fin is 0.7896, but is not significant, indicating that financialization may not have a significant impact on stakeholder-related CSR activities. In Column (2), the coefficient of Competition is -1.6915, significant at the 10% level, demonstrating that there is a negative association between product market competition and stakeholder-related CSR activities. In Column (3), financialization cannot have a significant impact on stakeholder-related CSR activities, while product market competition can promote this kind of CSR engagements. After introducing control variables, the results of Columns (4)–(6) can support that product market competition can reduce the preferences of managers for stakeholder-related CSR activities. Column (7) examines the impact of product market competition on the association between financialization and stakeholder-related CSR engagements. Based on the result of Column (7), Competition cannot significantly strengthen the impact of Fin on CSR_Stakeholder, indicating that competitive environment may not resolve the conflicts between stakeholders and shareholders.

**Table 5:** The baseline results of stakeholder-related CSR engagements.

Variables	CSR_Stakeholder						
	(1)	(2)	(3)	(4)	(5)	(6)	(7)
Fin	0.7896		0.6060	2.0442		1.8276	1.8743
	(0.51)		(0.38)	(1.34)		(1.19)	(1.22)
Competition		-1.6915*	-1.6773*		-1.9962**	-1.9530**	-1.9752**
		(-1.87)	(-1.85)		(-2.37)	(-2.30)	(-2.32)
Fin * Competition							5.7184
							(1.18)
Size				2.3670***	2.3737***	2.3785***	2.3772***
				(8.82)	(8.90)	(8.89)	(8.89)
Sales				-0.3428	-0.3715	-0.3661	-0.3681
				(-1.12)	(-1.20)	(-1.18)	(-1.20)
First				5.2895***	5.1896***	5.2478***	5.2551***
				(3.21)	(3.16)	(3.19)	(3.20)
Independ				15.5059**	15.3853**	15.5979**	15.3077**
				(2.55)	(2.54)	(2.57)	(2.51)
Director				4.9815**	4.8250**	4.9107**	4.7843**
				(2.56)	(2.48)	(2.53)	(2.45)
Dual				-0.5750	-0.6304	-0.5998	-0.6069
				(-1.27)	(-1.40)	(-1.32)	(-1.34)
Subsidy				0.0076	-1.4322	-1.7626	-1.0721
				(0.00)	(-0.11)	(-0.13)	(-0.08)
Constant	14.1798***	15.1619***	15.1179***	-55.0636***	-53.5320***	-54.0619***	-53.6912***
	(7.91)	(8.17)	(8.15)	(-6.88)	(-6.73)	(-6.77)	(-6.71)
Year	Yes	Yes	Yes	Yes	Yes	Yes	Yes
Industry	Yes	Yes	Yes	Yes	Yes	Yes	Yes
Observations	2116	2116	2116	2116	2116	2116	2116
R^2^	0.0835	0.0850	0.0851	0.1627	0.1644	0.1648	0.1652

Note: ***, **, * represent the significance at the level of 1%, 5%, 10% respectively; t statistics are in parentheses.

### Heterogeneity test

#### The test of ownership

The ownership of sports goods manufacturing firms can determine the external resources and the support provided by the government. Non-state-owned enterprises (non-SOEs) are more dependent on investors, and have some disadvantages in environmental policies. State-owned enterprises (SOEs) can get more support from local government, and their development strategies may have a direct impact on suppliers and consumers. In this situation, this study divides sports goods manufacturing firms into two subsamples, including the subsample of non-SOEs and the subsample of SOEs. The results of different ownership are reported in Table 6.

**Table 6:** The results of different ownership.

Variables	CSR_Shareholder			CSR_Stakeholder		CSR_Shareholder			CSR_Stakeholder	
	non-SOEs					SOEs				
	(1)	(2)	(3)		(4)	(5)	(6)	(7)		(8)
Fin	4.0130***	4.1965***		4.2150***	4.2798***	4.6970**	4.6228**		-13.8065***	-13.8433***
	(3.21)	(3.56)		(2.64)	(2.68)	(2.24)	(2.24)		(-3.11)	(-3.10)
Competition	1.3102**	1.2466**		-1.0869	-1.1093	-0.6570	-0.6661		-4.0182	-4.0227
	(2.37)	(2.28)		(-1.41)	(-1.44)	(-0.77)	(-0.78)		(-1.61)	(-1.61)
Fin * Competition		14.6533***			5.1688		5.1061			2.5324
		(4.27)			(1.09)		(0.74)			(0.18)
Size	0.9375***	0.9407***		1.9217***	1.9228***	0.7870***	0.7768***		2.3176***	2.3125***
	(6.47)	(6.51)		(6.49)	(6.50)	(3.31)	(3.28)		(3.86)	(3.83)
Sales	-0.3146	-0.2887		-0.1395	-0.1303	-0.6886**	-0.7238**		-1.1134	-1.1308
	(-1.24)	(-1.13)		(-0.43)	(-0.41)	(-2.25)	(-2.33)		(-1.63)	(-1.63)
First	10.8858***	10.9043***		2.8975*	2.9040*	8.1417***	8.1529***		8.6830*	8.6886*
	(10.16)	(10.23)		(1.83)	(1.84)	(4.05)	(4.05)		(1.72)	(1.72)
Independ	7.6057**	6.5102*		15.0004***	14.6140**	-1.7960	-1.5084		9.2335	9.3761
	(2.10)	(1.81)		(2.60)	(2.52)	(-0.27)	(-0.23)		(0.50)	(0.51)
Director	3.1656***	2.8876**		1.8747	1.7766	2.6143	2.4091		11.1090**	11.0072**
	(2.79)	(2.57)		(1.01)	(0.95)	(1.38)	(1.23)		(2.07)	(2.03)
Dual	0.5109*	0.4863		-0.4622	-0.4708	-1.1154	-1.0981		0.9719	0.9805
	(1.65)	(1.58)		(-1.05)	(-1.07)	(-1.41)	(-1.38)		(0.45)	(0.46)
Subsidy	0.0006	1.6169		-5.1137	-4.5436	-7.8242	-7.2925		1.1643	1.4280
	(0.00)	(0.22)		(-0.44)	(-0.38)	(-0.64)	(-0.60)		(0.03)	(0.04)
Constant	-20.6821***	-19.9324***		-37.8701***	-37.6057***	-16.0224***	-15.4298**		-63.3457***	-63.0518***
	(-4.84)	(-4.72)		(-4.30)	(-4.27)	(-2.71)	(-2.56)		(-3.71)	(-3.64)
Year	Yes	Yes		Yes	Yes	Yes	Yes		Yes	Yes
Industry	Yes	Yes		Yes	Yes	Yes	Yes		Yes	Yes
Observations	1622	1622		1622	1622	494	494		494	494
R^2^	0.1466	0.1545		0.1536	0.1540	0.2533	0.2541		0.2429	0.2430

Note: ***, **, * represent the significance at the level of 1%, 5%, 10% respectively; t statistics are in parentheses.

In Table 6, Columns (1)–(4) respectively explore the impact of financialization and product market competition on CSR engagements in the subsample of non-SOEs. In the results of Columns (1) and (2), there is a positive association between financialization and shareholder-related CSR engagements, as well as product market competition. Moreover, product market competition can strengthen the impact of financialization on the shareholder-related CSR engagements of non-SOEs. In the results of Columns (3) and (4), there is a positive association between financialization and stakeholder-related CSR engagements, but the impact of product market competition is not significant. For sports goods manufacturing firms, the managers of non-SOEs will pay attention to the well-being of shareholders and stakeholders, and they prefer to invest in financial assets to balance the relationship between shareholders and stakeholders. Columns (5)–(8) respectively explore the impact of financialization and product market competition on CSR engagements in the subsample of SOEs. In the results of Columns (5) and (6), there is a positive association between financialization and shareholder-related CSR engagements, but the impact of product market competition is not significant. In the results of Columns (7) and (8), there is a negative association between financialization and stakeholder-related CSR engagements, while the impact of product market competition is also not significant. Combined with the results of Columns (5)–(8), it can be found that the investments in financial assets will change the preference of SOEs’ managers in different CSR engagements, making them may pay more attention to the well-being of shareholders than those of stakeholders.

**The test of high-polluting industry.** Considering the fact that sports goods manufacturing firms may bring in air pollution or other environmental violations, especially for firms belonging to high-polluting industries. From this view, high-polluting firms will face some constraints from external factors, including environmental policies, public concerns, and energy limitations. In this situation, this study divides sports goods manufacturing firms into two subsamples, including the subsample in non-high-polluting industries and the subsample in high-polluting industries. The results of non-high-polluting and high-polluting industries are reported in Table 7.

**Table 7:** The results of non-high-polluting and high-polluting industries.

Variables	CSR_Shareholder		CSR_Stakeholder		CSR_Shareholder		CSR_Stakeholder	
	Non-High-polluting industries				High-polluting industries			
	(1)	(2)	(3)	(4)	(5)	(6)	(7)	(8)
Fin	5.6571***	5.5642***	6.1965***	6.1719***	1.3206	1.5356	-3.9371*	-3.9002*
	(3.91)	(4.12)	(2.99)	(2.97)	(0.74)	(0.89)	(-1.80)	(-1.77)
Competition	1.1570**	1.0413*	-2.4425**	-2.4731**	0.6104	0.6259	-1.4148	-1.4122
	(2.03)	(1.83)	(-2.17)	(-2.20)	(0.77)	(0.79)	(-1.08)	(-1.08)
Fin * Competition		17.8908***		4.7316		8.6798*		1.4896
		(4.43)		(0.71)		(1.74)		(0.22)
Size	0.8433***	0.8603***	2.3978***	2.4023***	0.4206**	0.4033*	2.3167***	2.3137***
	(5.50)	(5.59)	(7.24)	(7.24)	(2.05)	(1.96)	(5.25)	(5.22)
Sales	-0.9178***	-0.9840***	0.0581	0.0406	0.1945	0.2659	-1.5132***	-1.5009**
	(-3.66)	(-3.91)	(0.16)	(0.11)	(0.52)	(0.70)	(-2.63)	(-2.57)
First	11.4729***	11.3307***	2.0098	1.9722	7.8579***	7.9708***	8.8758***	8.8951***
	(9.21)	(9.12)	(0.98)	(0.96)	(5.30)	(5.40)	(3.44)	(3.43)
Independ	-2.0460	-2.9619	18.7821**	18.5399**	12.0443**	11.6202**	16.8837*	16.8110*
	(-0.54)	(-0.79)	(2.31)	(2.26)	(2.40)	(2.30)	(1.87)	(1.86)
Director	1.8099	1.2772	8.3431***	8.2022***	1.9865	1.9072	1.0785	1.0649
	(1.54)	(1.09)	(3.22)	(3.14)	(1.27)	(1.22)	(0.35)	(0.35)
Dual	1.3150***	1.2708***	0.3519	0.3402	0.5094	0.5196	-2.2518***	-2.2500***
	(3.54)	(3.44)	(0.60)	(0.58)	(1.15)	(1.17)	(-3.11)	(-3.11)
Subsidy	-0.5081	4.8905	38.3697*	39.7974*	-18.7097	-19.8285	-37.9445	-38.1365
	(-0.04)	(0.41)	(1.71)	(1.79)	(-1.51)	(-1.61)	(-1.49)	(-1.50)
Constant	-14.5380***	-13.4101***	-64.4684***	-64.1701***	-9.0488	-8.5081	-42.6408***	-42.5480***
	(-3.47)	(-3.24)	(-6.15)	(-6.11)	(-1.48)	(-1.39)	(-3.49)	(-3.47)
Year	Yes	Yes	Yes	Yes	Yes	Yes	Yes	Yes
Industry	Yes	Yes	Yes	Yes	Yes	Yes	Yes	Yes
Observations	1205	1205	1205	1205	911	911	911	911
R^2^	0.1389	0.1504	0.1505	0.1508	0.1424	0.1449	0.2220	0.2220

Note: ***, **, * represent the significance at the level of 1%, 5%, 10% respectively; t statistics are in parentheses.

In Table 7, Columns (1)–(4) respectively explore the impact of financialization and product market competition on the CSR engagements of sports goods manufacturing firms in non-high-polluting industries. In the results of Columns (1) and (2), there is a positive association between financialization and shareholder-related CSR engagements, as well as product market competition. Moreover, product market competition can strengthen the impact of financialization on the shareholder-related CSR engagements of non-high-polluting firms. In the results of Columns (3) and (4), there is a positive association between financialization and stakeholder-related CSR engagements, but product market competition may have a negative impact on this kind of CSR activities. For non-high-polluting firms, managers will invest more in financial assets to resolve the conflicts between shareholders and stakeholders, while the pressure from competition may reduce their attention on stakeholders. Columns (5)–(8) respectively explore the impact of financialization and product market competition on the CSR engagements of sports goods manufacturing firms in high-polluting industries. In the results of Columns (5) and (6), there is no significant association between financialization and shareholder-related CSR engagements, as well as product market competition. In the results of Columns (7) and (8), there is a negative association between financialization and stakeholder-related CSR engagements, while the impact of product market competition is not significant. It is interesting that the managers of sports goods manufacturing firms in high-polluting industries may not care about the well-being of shareholders, and the investments in financial assets will motivate them to ignore the well-being of stakeholders.

#### The test of financial constraint

The CSR engagements of sports goods manufacturing firms can represent the efforts of such firms in meeting the expectations of the society. However, these activities may not produce economic or environmental benefits in the short run, and could also lead to a shortage of funds. In this situation, this study adopts the SA-index to show the degree of financial constraints for sports goods manufacturing firms [49]. If the SA-index of sports goods manufacturing firms is below the mean of overall sample, we will set these firms as the subsample of high financial constraints. If the SA-index of sports goods manufacturing firms is above the mean of overall sample, we will set these firms as the subsample of low financial constraints. The results of different financial constraints are reported in Table 8.

**Table 8:** The results of different financial constraints.

Variables	CSR_Shareholder		CSR_Stakeholder		CSR_Shareholder		CSR_Stakeholder	
	High constraints				Low constraints			
	(1)	(2)	(3)	(4)	(5)	(6)	(7)	(8)
Fin	1.2952	1.7028	0.3465	0.3568	8.5297***	8.5778***	2.7383	2.3807
	(0.87)	(1.20)	(0.23)	(0.24)	(5.52)	(5.56)	(0.91)	(0.78)
Competition	1.6971**	1.4759**	-1.3497	-1.3553	-0.3563	-0.3770	-2.2980	-2.1438
	(2.49)	(2.17)	(-1.43)	(-1.40)	(-0.55)	(-0.58)	(-1.63)	(-1.51)
Fin * Competition		13.6388***		0.3438		-1.4502		10.7870
		(3.95)		(0.07)		(-0.26)		(0.84)
Size	0.0386	0.0678	1.1276**	1.1283**	0.3361*	0.3378*	3.6621***	3.6488***
	(0.10)	(0.18)	(2.43)	(2.44)	(1.71)	(1.73)	(7.25)	(7.21)
Sales	-0.2387	-0.2289	0.1391	0.1393	-0.8625***	-0.8633***	-0.9520**	-0.9463**
	(-0.64)	(-0.62)	(0.32)	(0.32)	(-3.36)	(-3.37)	(-2.07)	(-2.08)
First	12.3947***	12.2626***	8.5010***	8.4977***	7.0046***	6.9934***	1.8504	1.9339
	(7.46)	(7.38)	(3.93)	(3.94)	(6.24)	(6.25)	(0.79)	(0.83)
Independ	11.2911**	9.5945*	14.5812*	14.5384*	-0.3779	-0.3730	13.6027	13.5665
	(2.26)	(1.93)	(1.95)	(1.93)	(-0.10)	(-0.10)	(1.56)	(1.55)
Director	4.6074***	3.8896**	3.4479	3.4298	-0.2766	-0.2787	4.9641*	4.9800*
	(2.93)	(2.47)	(1.45)	(1.44)	(-0.25)	(-0.25)	(1.80)	(1.81)
Dual	1.4239***	1.3920***	-1.5085***	-1.5093***	0.3432	0.3427	0.0444	0.0476
	(3.53)	(3.46)	(-2.79)	(-2.79)	(0.84)	(0.84)	(0.06)	(0.06)
Subsidy	-6.1989	-5.6480	2.9092	2.9231	-11.1945	-11.6731	-36.2768	-32.7163
	(-0.87)	(-0.81)	(0.20)	(0.20)	(-0.37)	(-0.38)	(-0.66)	(-0.60)
Constant	-10.3593	-8.6922	-27.2318**	-27.1898**	4.5274	4.5339	-77.1342***	-77.1825***
	(-1.16)	(-0.98)	(-2.25)	(-2.24)	(0.90)	(0.91)	(-5.69)	(-5.70)
Year	Yes	Yes	Yes	Yes	Yes	Yes	Yes	Yes
Industry	Yes	Yes	Yes	Yes	Yes	Yes	Yes	Yes
Observations	1058	1058	1058	1058	1058	1058	1058	1058
R^2^	0.1409	0.1494	0.1110	0.1110	0.1357	0.1358	0.2225	0.2230

Note: ***, **, * represent the significance at the level of 1%, 5%, 10% respectively; t statistics are in parentheses.

In Table 8, Columns (1)–(4) respectively explore the impact of financialization and product market competition on the CSR engagements of sports goods manufacturing firms with high financial constraints. In the results of Columns (1) and (2), there is a positive association between product market competition and shareholder-related CSR engagements, and competition can strengthen the impact of financialization on the shareholder-related CSR engagements of firms with high financial constraints. In the results of Columns (3) and (4), there is no significant association between financialization and stakeholder-related CSR engagements, as well as product market competition. For the firms with high financial constraints, the pressure from product market competition can motivate managers to pay more attention to the well-being of shareholders, who can provide external funds. Columns (5)–(8) respectively explore the impact of financialization and product market competition on the CSR engagements of sports goods manufacturing firms with low financial constraints. In the results of Columns (5) and (6), there is a positive association between financialization and shareholder-related CSR engagements, but the impact of product market competition is not significant. In the results of Columns (7) and (8), there is no significant association between financialization and stakeholder-related CSR engagements, as well as product market competition. Combined with the results of Columns (5)–(8), the managers of firms with low financial constraints may prefer to invest in financial assets to obtain short-term profits, so as to meet the expectations of shareholders.

#### The test of top manager compensation

According to the principal-agent theory, the conflicts between owner and manager are mainly caused by the interests of agents. Managers’ motivation to get more interests will influence the well-being of shareholders. In order to improve the management efficiency of managers, most firms will pay them high compensation. Therefore, this study divides sports goods manufacturing firms into two subsamples, including the subsample of low compensation and the subsample of high compensation. If the compensation of managers is below the mean of overall sample, we will set these firms as the subsample of low compensation. If the compensation of managers is above the mean of overall sample, we will set these firms as the subsamples of high compensation. The results of top manager compensation are reported in Table 9.

**Table 9:** The results of top manager compensation.

Variables	CSR_Shareholder		CSR_Stakeholder		CSR_Shareholder		CSR_Stakeholder	
	Low compensation				High compensation			
	(1)	(2)	(3)	(4)	(5)	(6)	(7)	(8)
Fin	0.2744	1.1508	-0.6201	-0.4688	7.0338***	6.7756***	3.8798*	3.4487
	(0.18)	(0.78)	(-0.33)	(-0.25)	(4.32)	(4.07)	(1.67)	(1.39)
Competition	0.4528	0.3877	-2.4831**	-2.4943**	1.4439*	1.4517*	-1.4230	-1.4100
	(0.78)	(0.67)	(-2.55)	(-2.54)	(1.88)	(1.89)	(-0.98)	(-0.97)
Fin * Competition		13.6433***		2.3559		5.0960		8.5091
		(3.87)		(0.44)		(0.81)		(0.79)
Size	0.0601	0.0642	1.4343***	1.4351***	0.3106*	0.3101*	2.5721***	2.5713***
	(0.31)	(0.33)	(3.44)	(3.45)	(1.84)	(1.83)	(7.23)	(7.23)
Sales	-0.5219*	-0.5546**	0.3200	0.3143	-0.2655	-0.2616	-0.8081*	-0.8015*
	(-1.85)	(-2.00)	(0.71)	(0.71)	(-0.92)	(-0.90)	(-1.88)	(-1.87)
First	11.6921***	11.6548***	8.6189***	8.6124***	7.4639***	7.4754***	2.6906	2.7098
	(8.56)	(8.54)	(3.85)	(3.85)	(5.84)	(5.87)	(1.16)	(1.17)
Independ	4.1290	2.6755	15.7352**	15.4842**	0.6017	0.5697	10.9530	10.8997
	(0.94)	(0.61)	(2.11)	(2.07)	(0.15)	(0.14)	(1.25)	(1.25)
Director	2.4712*	1.8257	3.0839	2.9724	-1.4622	-1.4560	4.1618	4.1721
	(1.90)	(1.40)	(1.37)	(1.31)	(-1.09)	(-1.09)	(1.45)	(1.45)
Dual	1.6956***	1.6917***	-1.1603**	-1.1610**	-0.1958	-0.2036	-0.2155	-0.2286
	(4.38)	(4.38)	(-2.03)	(-2.04)	(-0.47)	(-0.49)	(-0.31)	(-0.33)
Subsidy	-5.7439	-4.5666	4.0493	4.2526	-3.6912	-2.5372	-73.6138	-71.6869
	(-0.72)	(-0.62)	(0.26)	(0.28)	(-0.12)	(-0.08)	(-1.35)	(-1.33)
Constant	-2.2768	-0.5590	-33.0080***	-32.7114***	7.5357	7.4335	-50.6308***	-50.8015***
	(-0.43)	(-0.10)	(-2.87)	(-2.83)	(1.51)	(1.49)	(-4.53)	(-4.54)
Year	Yes	Yes	Yes	Yes	Yes	Yes	Yes	Yes
Industry	Yes	Yes	Yes	Yes	Yes	Yes	Yes	Yes
Observations	1058	1058	1058	1058	1058	1058	1058	1058
R^2^	0.1438	0.1534	0.1325	0.1326	0.1100	0.1105	0.2263	0.2267

Note: ***, **, * represent the significance at the level of 1%, 5%, 10% respectively; t statistics are in parentheses.

In Table 9, Columns (1)–(4) respectively explore the impact of financialization and product market competition on the CSR engagements of sports goods manufacturing firms with low compensation. In the results of Columns (1) and (2), there is no significant association between financialization and shareholder-related CSR engagements, as well as product market competition. In the results of Columns (3) and (4), there is a negative association between product market competition and stakeholder-related CSR engagements, but the impact of financialization is not significant. For the firms with low compensation, the pressure of product market will motivate managers to pay more attention to their interests and ignore the well-being of stakeholders. Columns (5)–(8) respectively explore the impact of financialization and product market competition on the CSR engagements of sports goods manufacturing firms with high compensation. In the results of Columns (5) and (6), there is a positive association between financialization and shareholder-related CSR engagements, as well as product market competition. In the results of Columns (7) and (8), there is a positive association between financialization and stakeholder-related CSR engagements, while product market competition may weaken the impact of financialization on such CSR activities. The results of Columns (5)–(8) demonstrate that the strategy of compensation for top managers can mitigate the conflicts between owners and managers, which will help managers to focus on different CSR activities.

#### Robustness test

When sports goods manufacturing firms adopt different CSR engagements during their operations, they need to rely on internal and external resources to implement some CSR activities. Therefore, the driving factors of CSR engagements may have a long-term impact on the managers’ adoption of different CSR activities. In this situation, we make the variables of financialization, product market competition and interaction term lag one year respectively, and examine the long-term impact of financialization and competition on CSR engagements. The results of lagged independent variables are reported in Table 10.

**Table 10:** The results of lagged independent variables.

Variables	CSR_Shareholder		CSR_Stakeholder	
	(1)	(2)	(3)	(4)
Fin_t-1_	3.5285**	3.8302**	2.4285	2.5219
	(2.27)	(2.56)	(1.24)	(1.28)
Competition_t-1_	1.7616**	1.7889**	-2.5780**	-2.5695**
	(2.49)	(2.55)	(-2.31)	(-2.31)
Fin_t-1_ * Competition_t-1_		11.6283***		3.5999
		(2.95)		(0.55)
Size	0.9761***	0.9781***	2.2052***	2.2059***
	(5.44)	(5.46)	(5.55)	(5.55)
Sales	-0.1612	-0.1403	-0.3591	-0.3526
	(-0.58)	(-0.50)	(-1.02)	(-1.00)
First	11.0971***	11.1850***	4.8008**	4.8280**
	(7.84)	(7.95)	(2.00)	(2.00)
Independ	1.6957	0.9784	-1.3935	-1.6156
	(0.36)	(0.21)	(-0.20)	(-0.23)
Director	1.4834	0.9690	1.2117	1.0525
	(1.11)	(0.72)	(0.48)	(0.41)
Dual	0.8264*	0.7755*	-0.1317	-0.1475
	(1.95)	(1.83)	(-0.20)	(-0.22)
Subsidy	-20.1323**	-15.5013*	4.8370	6.2707
	(-2.41)	(-1.77)	(0.43)	(0.54)
Constant	-17.8323***	-16.6403***	-35.7473***	-35.3783***
	(-3.44)	(-3.19)	(-3.18)	(-3.13)
Year	Yes	Yes	Yes	Yes
Industry	Yes	Yes	Yes	Yes
Observations	1031	1031	1031	1031
R^2^	0.1592	0.1638	0.1577	0.1579

Note: ***, **, * represent the significance at the level of 1%, 5%, 10% respectively; t statistics are in parentheses.

In Table 10, Columns (1) and (2) respectively explore the impact of financialization and product market competition on the shareholder-related CSR activities of sports goods manufacturing firms. It can be seen that there is a positive association between financialization and shareholder-related CSR activities, as well as product market competition. Moreover, product market competition can strengthen the impact of financialization on shareholder-related CSR activities. The results of Columns (1) and (2) support the baseline results provided in Table 4. Columns (3) and (4) respectively explore the impact of financialization and product market competition on the stakeholder-related CSR activities of sports goods manufacturing firms. In the results of Columns (3) and (4), there is a negative association between product market competition and stakeholder-related CSR activities, but the impact of financialization is not significant. The results of Columns (3) and (4) can also support the baseline results provided in Table 5.

In order to support the findings of empirical analysis, we change the measurement of competition, and use the Herfindahl-Hirschman Index (HHI) to measure the competition degree of firm *i*. In this process, the industry of each firm will be used to matched with the HHI of different industries. The results of alternative competition measurement are reported in Table 11.

**Table 11:** The results of alternative competition measurement.

Variables	CSR_Shareholder			CSR_Stakeholder		
	(1)	(2)	(3)	(4)	(5)	(6)
Fin	2.8768**	3.8354***	3.9197***	0.7476	2.0565	2.0672
	(2.41)	(3.40)	(3.47)	(0.48)	(1.35)	(1.33)
HHI	4.3037*	3.8042*	3.8007*	1.6998	-0.5086	-0.5090
	(1.78)	(1.66)	(1.66)	(0.43)	(-0.13)	(-0.13)
Fin*HHI			7.8663			1.0024
			(0.53)			(0.05)
Size		0.6589***	0.6517***		2.3673***	2.3664***
		(5.31)	(5.22)		(8.82)	(8.79)
Sales		-0.5707***	-0.5688***		-0.3412	-0.3409
		(-2.75)	(-2.74)		(-1.12)	(-1.12)
First		9.3783***	9.3688***		5.2972***	5.2960***
		(9.90)	(9.88)		(3.21)	(3.21)
Independ		3.3641	3.3884		15.5196**	15.5227**
		(1.12)	(1.12)		(2.56)	(2.56)
Director		1.5230	1.5513*		4.9806**	4.9842**
		(1.63)	(1.66)		(2.56)	(2.56)
Dual		0.9494***	0.9489***		-0.5799	-0.5800
		(3.31)	(3.31)		(-1.27)	(-1.27)
Subsidy		-8.6927	-8.8544		0.0045	-0.0161
		(-1.11)	(-1.13)		(0.00)	(-0.00)
Constant	11.3797***	-10.8685***	-10.7884***	13.9083***	-54.9950***	-54.9848***
	(14.02)	(-3.16)	(-3.13)	(7.27)	(-6.85)	(-6.84)
Year	Yes	Yes	Yes	Yes	Yes	Yes
Industry	Yes	Yes	Yes	Yes	Yes	Yes
Observations	2116	2116	2116	2116	2116	2116
R^2^	0.0501	0.1245	0.1246	0.0836	0.1627	0.1627

Note: ***, **, * represent the significance at the level of 1%, 5%, 10% respectively; t statistics are in parentheses.

In Table 11, Columns (1)–(3) respectively explore the impact of financialization and product market competition on the shareholder-related CSR activities of sports goods manufacturing firms. There is a positive association between HHI and shareholder-related CSR activities, demonstrating that the competition degree obtained by industry classification can capture the promotion of market competition to this kind of CSR engagements. Columns (4)—(6) respectively explore the impact of financialization and product market competition on the stakeholder-related CSR activities of sports goods manufacturing firms. There is a negative association between HHI and stakeholder-related CSR activities, but the coefficient of HHI is not significant. Although the Herfindahl-Hirschman Index can measure market competition based on industry classification, this measurement will not capture the competitive environment faced by individual firm. Moreover, there is no sports goods related industries, so the HHI of such firms may not have a direct impact on CSR engagements.

Sports goods manufacturers engaging in different CSR activities may be driven by various factors, such as manager characteristics, government support, industrial policy. In this situation, firms in sports goods manufacturing industries can choose different CSR engagements to meet the demand of shareholders or stakeholders. During the empirical analysis of CSR engagements, there is a self-selection bias in our empirical models. Therefore, we use the propensity score matching (PSM) method to address this issue. Based on the results of baseline test, competition can directly influence the CSR engagements of sports goods manufacturers. We define firms with Competition above the top quartile as the treatment group, while the other firms as the control group. In the matching process, the nearest neighbor method with replacement is used to obtain the propensity scores, and all control variables are utilized as matching criteria. The results of PSM test are reported in Table 12.

**Table 12:** The results of PSM test.

Variables	Treat1	CSR_Shareholder		Treat1	CSR_Stakeholder	
	(1)	(2)	(3)	(4)	(5)	(6)
Fin		3.806***	3.6155***		1.6744	1.5819
		(3.2)	(3.23)		(1.02)	(0.95)
Competition		1.0311*	0.9411*		-2.1079**	-2.1516**
		(1.95)	(1.79)		(-2.29)	(-2.32)
Fin * Competition			14.1779***			6.8852
			(4.33)			(1.33)
Size	0.0169	0.5665***	0.5705***	0.0169	2.4496***	2.4515***
	(0.35)	(4.14)	(4.15)	(0.35)	(8.15)	(8.17)
Sales	-0.1145	-0.7543***	-0.7405***	-0.1145	-0.0631	-0.0564
	(-1.22)	(-2.89)	(-2.83)	(-1.22)	(-0.17)	(-0.15)
First	0.3294	9.95***	9.9748***	0.3294	4.5999***	4.612***
	(0.9)	(9.08)	(9.14)	(0.9)	(2.58)	(2.59)
Independ	0.9808	4.3285	3.4427	0.9808	11.5408*	11.1106*
	(0.83)	(1.24)	(0.99)	(0.83)	(1.77)	(1.69)
Director	-0.7999**	1.452	1.0724	-0.7999**	4.3699**	4.1855*
	(-2.11)	(1.35)	(1.00)	(-2.11)	(2.02)	(1.93)
Dual	-0.1429	0.5644*	0.5485*	-0.1429	-0.6314	-0.6405
	(-1.28)	(1.7)	(1.64)	(-1.28)	(-1.25)	(-1.27)
Subsidy	-5.0356	-4.0474	0.8751	-5.0356	-40.5532	-38.1627
	(-0.83)	(-0.17)	(0.04)	(-0.83)	(-1.09)	(-1.02)
Constant	-0.0645	-8.8409**	-7.8904**	-0.0645	-51.5022***	-51.0407***
	(-0.05)	(-2.24)	(-2.01)	(-0.05)	(-5.69)	(-5.63)
Year	Yes	Yes	Yes	Yes	Yes	Yes
Industry	Yes	Yes	Yes	Yes	Yes	Yes
Observations	2116	1689	1689	2116	1689	1689
R^2^	0.047	0.1217	0.1293	0.047	0.1702	0.1708

Note: ***, **, * represent the significance at the level of 1%, 5%, 10% respectively; t statistics are in parentheses.

In Table 12, Columns (1)–(3) respectively explore the impact of financialization and product market competition on the shareholder-related CSR activities of sports goods manufacturing firms. Column (1) shows the first step’s regression results with Treat1 as dependent variable. After obtaining the successfully matched samples, Columns (2) and (3) show the second step’s regression results. There is a significantly positive association between financialization and shareholder-related CSR activities, as well as product market competition. Moreover, product market competition can still strengthen the impact of financialization on shareholder-related CSR activities. Columns (4)–(6) respectively explore the impact of financialization and product market competition on the stakeholder-related CSR activities of sports goods manufacturing firms. Column (4) shows the first step’s regression results with Treat1 as dependent variable. Columns (5) and (6) show the second step’s regression results based on the successfully matched samples. There is a significantly negative association between product market competition and shareholder-related CSR activities. However, the coefficient of financialization is not significant, as well as the interaction of financialization and competition.

In order to ensure the robustness of our findings, we employ a series of methodological tests, including the use of lagged independent variables, alternative measures of competition, and the Propensity Score Matching (PSM) method. Across these diverse tests, our baseline results remain consistent.

## Discussion

CSR engagements reflect firms’ dual objectives of achieving both economic and environmental benefits, while also addressing societal concerns. In developing economies, the performance of manufacturing firms is crucial for regional economic growth; however, their substantial contributions to pollution and energy consumption have raised public scrutiny and pose significant challenges to sustainable development. Sports goods manufacturers represent a unique segment within this sector. They often prioritize shareholder interests by focusing on production volume, sometimes at the expense of product quality. Conversely, regulatory bodies require these firms to undertake socially responsible activities to enhance stakeholder well-being. Given the notable environmental impact of sports goods manufacturers, their CSR commitment is crucial for aligning operations with investor and societal expectations.

Empirical analysis reveals that managers of sports goods manufacturing firms are often driven to prioritize shareholder benefits due to pressures to maximize profits and maintain competitiveness. This focus amplifies managers’ tendencies to invest in financial assets and implement initiatives that fulfill shareholder expectations. Unfortunately, this shareholder-related approach can overshadow stakeholder well-being and consequently attenuate stakeholder-related CSR engagements. In such firms, the divergent CSR engagements reflect the extant tension between shareholders and stakeholders. Interestingly, sports goods manufacturers may strategically employ CSR as a tool to achieve mutually beneficial (‘win-win’) outcomes, consistent with the framework suggested by Benabou and Tirole [19]. Moreover, firms operating in non-high-polluting industries exhibit different CSR engagements. When juxtaposed against stakeholder-related CSR engagements, product market competition exerts pressure on undercapitalized firms to attend more to shareholder benefits. Concurrently, financialization drives adequately funded firms towards similar prioritization, a trend that diverges from the findings of Cheng et al. [22]. Based on principal-agent theory, highly compensated managers tend to focus on shareholder-related CSR, thereby reducing agency conflicts. This study highlights the varied CSR engagements in sports goods manufacturing, emphasizing the significant role of financialization and competition in shaping CSR strategies.

## Conclusion and recommendation

### Conclusion

CSR engagements are crucial for enhancing societal well-being and environmental sustainability, especially in manufacturing industries. For sports goods manufacturing firms, characterized by smaller scales and lower profit margins, choosing the right CSR engagements is vital for aligning with their business models and product offerings. A deeper understanding of the factors driving CSR engagements can help these firms better balance shareholder and stakeholder interests, thereby enhancing organizational sustainability.

This study employs a text-based method to identify sports goods manufacturing firms in the Chinese market and assesses their competitive environments. The trend towards financialization is often adopted by managers to increase shareholder value. Within the unique context of the sports goods sector, managers implement varying strategies to satisfy either shareholder or stakeholder expectations. Our findings indicate a positive correlation between financialization, market competition, and shareholder-related CSR engagements, with competition amplifying the effects of financialization. Conversely, there is a negative relationship between market competition and stakeholder-related CSR engagements. Non-state-owned enterprises tend to be more responsive to financialization and competition in their CSR activities, whereas state-owned enterprises show lesser engagement with stakeholder welfare. In high-polluting industries, competition critically influences CSR adoption, particularly among less-polluting firms, while a focus on financial assets can neglect stakeholder interests. Financial constraints also moderate the impact of financialization and competition on shareholder-related CSR engagements. Additionally, managerial compensation can help balance the interests of shareholders and stakeholders, with higher pay potentially reducing agency conflicts.

This study discusses the driving forces behind CSR engagements among sports goods manufacturing firms, particularly in the Chinese market. By focusing on financial assets and the competitive environment, our research illuminates how sports goods manufacturers may stray from their fundamental objectives through CSR strategies. Importantly, our findings offer insights into mitigating conflicts between shareholders and stakeholders, thereby contributing to the sustainable development of these firms.

### Recommendation

The theoretical and empirical analyses conducted in this study underscore the pivotal role of CSR engagements for sports goods manufacturing firms. These firms can formulate appropriate strategies to achieve their development goals, and these strategies are also linked to different CSR engagements. The study has several implications:

First, managers of sports goods manufacturing firms must allocate greater attention to stakeholder well-being. Amid conflicts between shareholder interests and stakeholder needs, many firms prioritize investments in financial assets to maximize short-term profits. However, these firms also contribute to environmental issues and rely on value chains involving suppliers and consumers. Therefore, the adoption of appropriate CSR engagements is essential for mitigating shareholder-stakeholder conflicts.

Second, sports goods manufacturing firms should refocus on their core objectives, which revolve around the production of quality sports goods. Excessive financialization aimed at short-term profit enhancement can divert crucial resources from product development, thereby eroding competitive advantage. Thus, stakeholder-related CSR engagements, such as technological innovation and product updates, emerge as vital strategies for the sustained growth of these firms.

## Supporting information

S1 Data (DTA)

## References

[1] Yuan B, Xiang Q. Environmental regulation, industrial innovation and green development of Chinese manufacturing: Based on an extended CDM model. J Clean Prod. 2018;176: 895–908. doi: 10.1016/j.jclepro.2017.12.034

[2] Cheng J, Liu Y. The effects of public attention on the environmental performance of high-polluting firms: Based on big data from web search in China. J Clean Prod. 2018;186: 335–341. doi: 10.1016/j.jclepro.2018.03.146

[3] Tsai FS. When and how group diversity facilitate innovativeness? The roles of knowledge heterogeneity and governance. Knowl Manag Res Pract. 2021. doi: 10.1080/14778238.2021.2004950

[4] Dowling M, Robinson L, Washington M. Taking advantage of the London 2012 Olympic Games: corporate social responsibility through sport partnerships. Eur Sport Manag Q. 2013;13: 269–292. doi: 10.1080/16184742.2013.774039

[5] Robertson J, Eime R, Westerbeek H. The social responsibilities of sport governing bodies and the role of sport governance. Routledge Handbook of Sport Governance. 2019. pp. 381–394. doi: 10.4324/9780429440250-27

[6] Walzel S, Robertson J, Anagnostopoulos C. Corporate social responsibility in professional team sports organizations: An integrative review. J Sport Manag. 2018;32: 511–530. doi: 10.1123/jsm.2017-0227

[7] Aguinis H, Glavas A. What We Know and Don’t Know About Corporate Social Responsibility. J Manage. 2012;38: 932–968. doi: 10.1177/0149206311436079

[8] Havlinova A, Kukacka J. Corporate Social Responsibility and Stock Prices After the Financial Crisis: The Role of Strategic CSR Activities. J Bus Ethics. 2021. doi: 10.1007/s10551-021-04935-9

[9] Huang C, Chen Y. How to Enhance the Green Innovation of Sports Goods? Micro- and Macro-Level Evidence From China’s Manufacturing Enterprises. Front Environ Sci. 2022;9. doi: 10.3389/fenvs.2021.809156

[10] Jäger J, Fifka M. The relationship between perceived corporate social responsibility and perceived organisational performance in professional sports organisations. Eur Sport Manag Q. 2022. doi: 10.1080/16184742.2022.2099922

[11] Ross WJ, Leopkey B. The adoption and evolution of environmental practices in the Olympic Games. Manag Sport Leis. 2017;22: 1–18. doi: 10.1080/23750472.2017.1326291

[12] Ross WJ, Leopkey B, Mercado HU. Governance of Olympic Environmental Stakeholders. J Glob Sport Manag. 2019;4: 331–350. doi: 10.1080/24704067.2018.1477524

[13] Robertson J, Karg A, Rowe K, Raw K. “My definition of community is community, and their definition is more around fan engagement”: balancing business and social logics of professional sport teams’ community activities. Sport Manag Rev. 2022. doi: 10.1080/14413523.2022.2087966

[14] Robertson J, Dowling M, Washington M, Leopkey B, Ellis DL, Smith L. Institutional Theory in Sport: A Scoping Review. J Sport Manag. 2022;36: 459–472. doi: 10.1123/jsm.2021-0179

[15] Tsai FS, Hsu IC. The effects of social capital on knowledge heterogeneity. Manag Decis. 2019;57: 1237–1253. doi: 10.1108/MD-12-2016-0909

[16] Wang J, Mao N. Does Financialization of Non-Financial Corporations Promote or Prohibit Corporate Risk-Taking? Emerg Mark Financ Trade. 2022;58: 1913–1924. doi: 10.1080/1540496X.2021.1944853

[17] Jin C, Tsai FS, Gu Q, Wu B. Does the porter hypothesis work well in the emission trading schema pilot? Exploring moderating effects of institutional settings. Res Int Bus Financ. 2022;62. doi: 10.1016/j.ribaf.2022.101732

[18] Zou HL, Zeng SX, Lin H, Xie XM. Top executives’ compensation, industrial competition, and corporate environmental performance: Evidence from China. Manag Decis. 2015;53: 2036–2059. doi: 10.1108/MD-08-2014-0515

[19] Benabou R, Tirole J. Individual and corporate social responsibility. Economica. 2010;77: 1–19. doi: 10.1111/j.1468-0335.2009.00843.x

[20] Li J, Wu D. Do corporate social responsibility engagements lead to real environmental, social, and governance impact? Manage Sci. 2020;66: 2564–2588. doi: 10.1287/mnsc.2019.3324

[21] Shahbaz M, Karaman AS, Kilic M, Uyar A. Board attributes, CSR engagement, and corporate performance: What is the nexus in the energy sector? Energy Policy. 2020;143. doi: 10.1016/j.enpol.2020.111582

[22] Cheng B, Ioannou I, Serafeim G. Corporate social responsibility and access to finance. Strateg Manag J. 2014;35: 1–23. doi: 10.1002/smj.2131

[23] Xie J, Nozawa W, Yagi M, Fujii H, Managi S. Do environmental, social, and governance activities improve corporate financial performance? Bus Strateg Environ. 2019;28: 286–300. doi: 10.1002/bse.2224

[24] Yuan Y, Lu LY, Tian G, Yu Y. Business Strategy and Corporate Social Responsibility. J Bus Ethics. 2020;162: 359–377. doi: 10.1007/s10551-018-3952-9

[25] Duran P, Heugens P, Van Essen M, Van Oosterhout H, Vishwanathan P. Strategic CSR: A Concept Building Meta‐Analysis. J Manag Stud. 2019;57: 314–350.

[26] Barko T, Cremers M, Renneboog L. Shareholder Engagement on Environmental, Social, and Governance Performance. J Bus Ethics. 2021. doi: 10.1007/s10551-021-04850-z

[27] Hillman AJ, Keim GD. Shareholder value, stakeholder management, and social issues: What’s the bottom line? Strateg Manag J. 2001;22: 125–139. doi: 10.1002/1097-0266(200101)22:2&lt;125::AID-SMJ150&gt;3.0.CO;2-H

[28] Xu J, Wei J, Lu L. Strategic stakeholder management, environmental corporate social responsibility engagement, and financial performance of stigmatized firms derived from Chinese special environmental policy. Bus Strateg Environ. 2019;28: 1027–1044. doi: 10.1002/bse.2299

[29] Karwowski M, Raulinajtys-Grzybek M. The application of corporate social responsibility (CSR) actions for mitigation of environmental, social, corporate governance (ESG) and reputational risk in integrated reports. Corp Soc Responsib Environ Manag. 2021;28: 1270–1284. doi: 10.1002/csr.2137

[30] Tang Z, Hull CE, Rothenberg S. How Corporate Social Responsibility Engagement Strategy Moderates the CSR-Financial Performance Relationship. J Manag Stud. 2012;49: 1274–1303. doi: 10.1111/j.1467-6486.2012.01068.x

[31] Soener M. Why do firms financialize? Meso-level evidence from the us apparel and footwear industry, 1991–2005. Socio-Economic Rev. 2015;13: 549–573. doi: 10.1093/ser/mwv006

[32] Knafo S, Dutta SJ. The myth of the shareholder revolution and the financialization of the firm. Rev Int Polit Econ. 2020;27: 476–499. doi: 10.1080/09692290.2019.1649293

[33] Xu X, Xuan C. A study on the motivation of financialization in emerging markets: The case of Chinese nonfinancial corporations. Int Rev Econ Financ. 2021;72: 606–623. doi: 10.1016/j.iref.2020.12.026

[34] Zhao XZ, Chen J, Chen FW, Wang W, Xia S. How high-polluting firms suffer from being distracted form intended purpose: A corporate social responsibility perspective. Int J Environ Res Public Health. 2020;17: 1–29. doi: 10.3390/ijerph17249197 33317027 PMC7764727

[35] Klinge TJ, Hendrikse R, Fernandez R, Adriaans I. Augmenting digital monopolies: A corporate financialization perspective on the rise of Big Tech. Compet Chang. 2022; 102452942211055. doi: 10.1177/10245294221105573

[36] Li S, Liu C, Peng X. The Impact of Financialization on Mergers and acquisitions:Evidence from Chinese Manufacturing Listed Firms. Emerg Mark Financ Trade. 2022. doi: 10.1080/1540496X.2022.2031969

[37] Destek MA, Manga M. Technological innovation, financialization, and ecological footprint: evidence from BEM economies. Environ Sci Pollut Res. 2021;28: 21991–22001. doi: 10.1007/s11356-020-11845-2 33411309

[38] Li F, Lundholm R, Minnis M. A Measure of Competition Based on 10-K Filings. J Account Res. 2013;51: 399–436. doi: 10.1111/j.1475-679X.2012.00472.x

[39] Lee JH, Byun HS, Park KS. Product market competition and corporate social responsibility activities: Perspectives from an emerging economy. Pacific Basin Financ J. 2018;49: 60–80. doi: 10.1016/j.pacfin.2018.04.001

[40] Zucchini L, Böhmer-Horländer S, Kretschmer T. Competitive pressure: competitive reactions at the group-level. Ind Innov. 2019;26: 643–666. doi: 10.1080/13662716.2018.1526666

[41] Martins HC. Competition and ESG practices in emerging markets: Evidence from a difference-in-differences model. Financ Res Lett. 2022;46. doi: 10.1016/j.frl.2021.102371

[42] Hull CE, Rothenberg S. Firm performance: The interactions of corporate social performance with innovation and industry differentiation. Strateg Manag J. 2008;29: 781–789. doi: 10.1002/smj.675

[43] Sheikh S. Corporate social responsibility and firm leverage: The impact of market competition. Res Int Bus Financ. 2019;48: 496–510. doi: 10.1016/j.ribaf.2018.11.002

[44] Dupire M M’Zali B. CSR Strategies in Response to Competitive Pressures. J Bus Ethics. 2018;148: 603–623. doi: 10.1007/s10551-015-2981-x

[45] Tsendsuren C, Yadav PL, Han SH, Kim H. Influence of product market competition and managerial competency on corporate environmental responsibility: Evidence from the US. J Clean Prod. 2021;304. doi: 10.1016/j.jclepro.2021.127065

[46] Huang B, Cui Y, Chan KC. Firm-level financialization: Contributing factors, sources, and economic consequences. Int Rev Econ Financ. 2022;80: 1153–1162. doi: 10.1016/j.iref.2022.04.007

[47] Leong CK, Yang YC. Market competition and firms’ social performance. Econ Model. 2020;91: 601–612. doi: 10.1016/j.econmod.2019.12.002

[48] Tang P, Yang S, Boehe D. Ownership and corporate social performance in China: Why geographic remoteness matters. J Clean Prod. 2018;197: 1284–1295. doi: 10.1016/j.jclepro.2018.06.288

[49] Hadlock CJ, Pierce JR. New evidence on measuring financial constraints: Moving beyond the KZ index. Rev Financ Stud. 2010;23: 1909–1940. doi: 10.1093/rfs/hhq009

[50] Tori D, Onaran Ö. The effects of financialization on investment: Evidence from firm-level data for the UK. Cambridge J Econ. 2018;42: 1393–1416. doi: 10.1093/CJE/BEX085

[51] Shi G, Sun J, Zhang L. Product market competition and earnings management: A firm-level analysis. J Bus Financ Account. 2018;45: 604–624. doi: 10.1111/jbfa.12300

[52] Hoberg G, Phillips G. Text-Based Network Industries and Endogenous Product Differentiation. J Polit Econ. 2016;124: 1423–1465. doi: 10.1086/688176

[53] Lee CMC, Ma P, Wang CCY. Search-based peer firms: Aggregating investor perceptions through internet co-searches. J financ econ. 2015;116: 410–431. doi: 10.1016/j.jfineco.2015.02.003

